# Fluid Overload and Renal Angina Index at Admission Are Associated With Worse Outcomes in Critically Ill Children

**DOI:** 10.3389/fped.2018.00118

**Published:** 2018-05-01

**Authors:** Sidharth K. Sethi, Veena Raghunathan, Shilpi Shah, Maninder Dhaliwal, Pranaw Jha, Maneesh Kumar, Sravanthi Paluri, Shyam Bansal, Maroun J. Mhanna, Rupesh Raina

**Affiliations:** ^1^Kidney and Urology Institute, Medanta, The Medicity Hospital, Gurgaon, India; ^2^Pediatric Intensive Care, Medanta, The Medicity Hospital, Gurgaon, India; ^3^Department of Pediatric Nephrology, Akron Children Hospital, Akron, OH, United States; ^4^Interfaith Medical Center, Brooklyn, NY, United States; ^5^Department of Pediatrics, Metrohealth Medical Center, Cleveland, OH, United States

**Keywords:** acute kidney injury, fluid overload, critical, oxygenation index, pediatrics

## Abstract

**Objectives:** We investigated the association of fluid overload and oxygenation in critically sick children, and their correlation with various outcomes (duration of ventilation, ICU stay, and mortality). We also assessed whether renal angina index (RAI) at admission can predict mortality or acute kidney injury (AKI) on day 3 after admission.

**Design and setting:** Prospective study, pediatric intensive care in a tertiary hospital.

**Duration:** June 2013-June 2014.

**Patients:** Patients were included if they needed invasive mechanical ventilation for >24 h and had an indwelling arterial catheter. Patients with congenital heart disease or those who received renal replacement therapy (RRT) were excluded.

**Methods:** Oxygenation index, fluid overload percent (daily, cumulative), RAI at admission and pediatric logistic organ dysfunction (PELOD) score were obtained in all critically ill children. KDIGO classification was used to define AKI, using both creatinine and urine output criteria. Admission data for determination of RAI included the use of vasopressors, invasive mechanical ventilation, percent fluid overload, and change in kidney function (estimated creatinine clearance). Univariable and multivariable approaches were used to assess the relations between fluid overload, oxygenation index and clinical outcomes. An RAI cutoff >8 was used to predict AKI on day 3 of admission and mortality.

**Results:** One hundred and two patients were recruited. Fluid overload predicted oxygenation index in all patients, independent of age, gender and PELOD score (*p* < 0.05). Fluid overload was associated with longer duration of ventilation (*p* < 0.05), controlled for age, gender, and PELOD score. Day-3 AKI rates were higher in patients with a RAI of 8 or more, and higher areas under the RAI curve had better prediction rates for Day-3 AKI. An RAI <8 had high negative predictive values (80–95%) for Day-3 AKI. RAI was better than traditional markers of pediatric severity of illness (PELOD) score for prediction of AKI on day 3.

**Conclusions:** This study emphasizes that positive fluid balance adversely affects intensive care in critically ill children. Further, the RAI prediction model may help optimize treatment and improve clinical prediction of AKI.

## Background

There are multiple reports of observational studies demonstrating a strong, independent association between fluid accumulation and poor clinical outcomes in children (1–7) and adults (8–13). The collective pediatric experience from multiple studies on critically ill children reveals that 10–20% fluid overload (FO) at continuous renal replacement therapy (CRRT) initiation confers a three- to eight-fold increased odds for mortality, after adjustment for illness severity, multi-organ failure, and age (from infants to young adults) (14). The largest study of pediatric populations included 297 patients from the Prospective Pediatric CRRT Registry (ppCRRT) Group, and showed that >20% FO is associated with higher odds of mortality compared with the presence of multiorgan failure (MOF) or oncological diagnosis at CRRT initiation (4). A recent study of critically ill children who were not on any form of renal replacement therapy (RRT) found that increasing fluid overload was associated with more worsening of oxygenation index in children (1).

Similar results have been seen in adult studies. The multicentre Program to Improve Care for Acute Renal Disease study showed an association between mortality and >10% fluid accumulation at RRT initiation (8). Another observational study on 212 adult patients with sepsis showed increased survival in sick patients who received both adequate initial fluid resuscitation and late conservative fluid management (10). The Fluid And Catheter Treatment Trial (FACTT) showed that a conservative fluid management strategy [using fluid restriction and diuretics to maintain lower central venous pressure and pulmonary capillary wedge pressure (PCWP)] led to fewer ventilator days, and suggested diuretic-induced negative fluid balance may improve survival in patients with acute kidney injury (AKI) (15).

There have been multiple reports that fluid overload is associated with impaired organ function (8, 9), respiratory morbidity (12, 16), ventilation days and length of stay in intensive care unit (ICU) (17, 18).

Identifying patients who are or are not at risk for severe and long lasting AKI in the pediatric ICU (PICU) is important for every PICU. An empiric clinical model of renal angina has been recently proposed to identify which critically ill patients would be at the greatest risk of AKI using patient demographic factors and early signs of injury, where presence of renal angina may delineate patients at higher risk for subsequent severe AKI (19–21). The injury parameters of Renal Angina Index (RAI) take into consideration a fall in estimated creatinine clearance from baseline or increase in intensive care fluid overload percentage.

In this prospective study, we investigated the association of fluid overload and oxygenation, and their correlation to duration of ventilation, ICU stay and mortality. We also assessed whether RAI using injury parameters (fall in creatinine clearance from baseline or increase in fluid overload or worse) can predict mortality and AKI on day 3.

## Patient and methods

### Design and setting

This was a prospective observational study done in a PICU in a tertiary hospital from June 2013 to 2014.

### Patients

All children (<18 years) who required invasive mechanical ventilation for >24 h and an indwelling arterial catheter were included in the study. Children with congenital heart disease or those who received RRT were excluded. We excluded congenital heart disease from the study, as the oxygenation index may not be truly reflective of the severity of illness, due to underlying complex cyanotic/acyanotic congenital heart disease. There are multiple studies showing association of fluid overload and worse outcomes in patients on RRT. However, there is lack of information on fluid overload in critically ill children, who are not on any form of RRT and their outcomes. Hence we specifically looked at this subset of patients.

Parental consent was taken from all children for enrolling in the study. The Institutional Review Board approved the study.

### Methods

Basic clinical data, oxygenation index, fluid overload percent (daily, cumulative), RAI, and PRISM score at admission and pediatric logistic organ dysfunction (PELOD) score were obtained in all critically ill children. Admission data for determination of RAI included the use of vasopressors; invasive mechanical ventilation; percent fluid overload and change in kidney function (estimated creatinine clearance).

Percent FO on Day 0 was determined by assessing the first 8 h of admission in the ICU on Day 0.The time frame of 8 h was felt to be beyond the generally accepted window of “early goal-directed therapy” (EGDT) of resuscitation (19). Daily fluid overload was calculated on each day of PICU stay taking into consideration all enteral and parenterally administered fluids including blood products and intravenous medications; and all fluid output including urinary, stool, and nasogastric outputs. The oxygenation index (OI) (mean airway pressure × Fio2 × 100/Pao2) was calculated daily as a surrogate measure of pulmonary dysfunction. The worst OI for a given day was calculated using the lowest Pao2, the mean airway pressure, and Fio2 recorded at that time.

### Statistical analysis

#### Baseline demographics

Continuous variables were reported as median with interquartile range and compared using the Mann–Whitney test. Categorical variables were summarized using frequency and proportion and compared by chi-square or Fisher's exact tests.

#### Fluid overload and oxygenation index (OI)

Peak FO% and peak OI were defined as the highest FO% and OI recorded during the study period, respectively. Stepwise multiple linear regression was used to evaluate the independent association between peak FO% and peak OI, controlling for confounders (*p*-value for exit was 0.2). Multiple linear regressions with generalized estimating equations were used to evaluate the repeated-measures daily association between FO% and daily OI, controlling for other daily confounding variables. Backward stepwise multiple logistic regression analysis was used to determine the association between peak FO% with mortality, controlling for age, gender, admission PELOD score, and admission OI (*p*-value for exit was 0.2). Multiple Cox regression was used to evaluate the independent effect of peak FO% on LOS (hospital and PICU) and length of mechanical ventilation to censor for death.

#### Relationship between FO% and OI

The association between these two measures were analyzed using multivariable analyses, controlling for confounders, such as age, gender, and admission PELOD score. The relationship between each study day's FO% and OI was done using repeated measures multivariable analyses, adjusting for the same potential confounders.

#### Renal angina index and risk of acute kidney injury on day 3

All patients were classified on Day 0 as fulfilling criteria for renal angina [i.e., being ANG(+) vs. ANG(–)] using the RAI. It has been previously shown by Basu et al., that An RAI score of >8 demonstrated the highest Youden's index and the highest negative predictive value and thus ANG(+) was defined as an RAI score >8 (19–21).

The primary outcome was the presence of severe AKI 72 h after PICU admission (Day-3 AKI), denoted as “subsequent severe AKI.”

An RAI cutoff of >8 was used to analyze the predictive performance of RAI (sensitivity, specificity, NPV, and PPV). Predictive performance of admission PRISM for AKI after 72 h was also tested. Multivariable regression was performed by comparing variables carrying univariable associations with the outcome and a *P* < 0.20. Area Under the Curve (AUC) AUC-values were calculated for each prediction model. In all analyses, a *p* < 0.05 was considered statistically significant.

## Results

### Descriptive statistics

**Figure 1:**
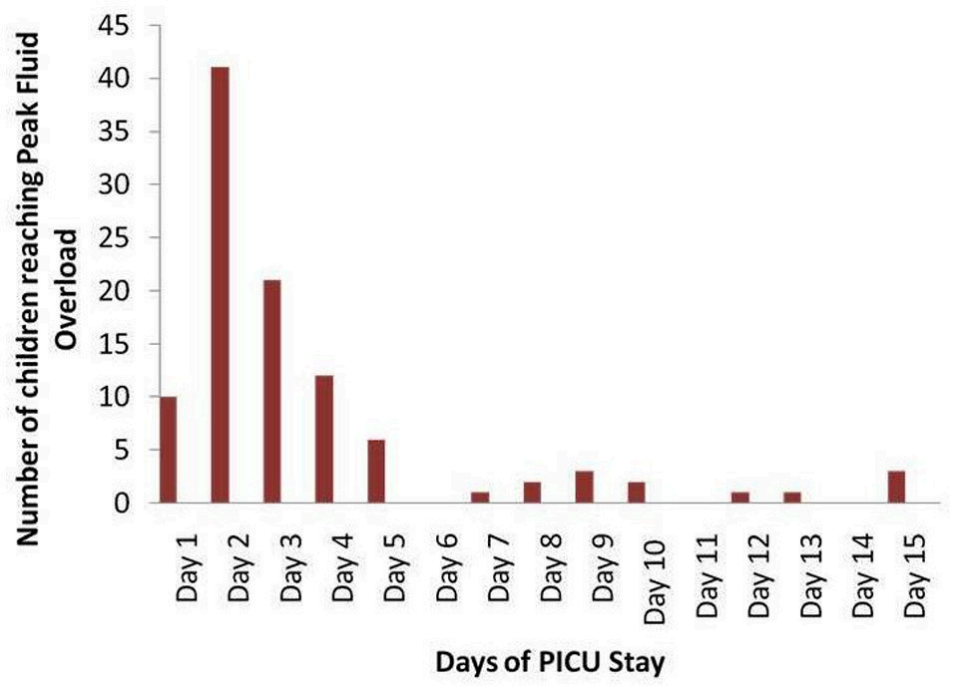
Number of children reaching peak fluid overload on each day of ICU admission.

One hundred and two children met the inclusion criteria. Mean age was 6.5 ± 5.9 months, and 67% were males (Table 1). Mean ± SD for PELOD score was 14.38 ± 10.86. Mean ICU stay was 9.1 ± 8.1 days and hospital stay was 12.9 ± 10 days. Maximum FO% was 8.7 ± 8.1 and maximum oxygenation index was 9.7 ± 10.8. Twenty-three children died during the stay (22.5%). The most common organ system dysfunction that led to admission was CNS (27.5%) followed by Liver/GIT (23.5%), sepsis (15.7%), and hemato-oncology (10.8%). The age break-up of the cohort was [<1 year-24 children (23.5%), 1–3 years-24 children (23.5%), 3–12 years-27 children (26.5%)] and 12–18 years-27 children (26.5%). Ninety percent of all children reached peak FO% on or before 5 days of PICU stay (Figure 1).

**Table 1:** Descriptive statistics of study parameters and outcomes.

Mean age (months) ± SD	6.5 ± 5.9
Males (%)	69 (67.6%)
Mean PELOD score ± SD	14.38 ± 10.86
Mean maximum fluid overload (FO) ± SD	8.7 ± 8.1
Mean maximum oxygenation index ± SD	9.7 ± 10.8
**OUTCOMES**	
Mean ICU stay (days) ± SD	9.1 ± 8.1
Mean ventilation days ± SD	5.72 ± 4.92
Mean hospital stay (days) ± SD	12.9 ± 10.0
Mortality (%)	23 (22.5%)

*PELOD Score**:** pediatric logistic organ dysfunction*.

### Fluid overload during admission and oxygenation index

For all days of ICU observation, multivariable analysis was done to determine association of FO% and OI. On every day of PICU stay, FO% was associated with OI, independent of age, gender and PELOD score (Table 2). The relationship between FO% and OI reached statistical significance when FO% reached more than 10%, and with a higher regression coefficient at FO% more than 15% (Table 3). On multivariable analysis, maximum FO% and PELOD score were significant predictors of peak OI (*p* = 0.04 and *p* = 0.002 respectively) (Table 4).

**Table 2:** Fluid overload during admission and oxygenation index.

**FO Day 1**	**Oxygenation index**
<5%	4.77 (3.93–5.60)
5–9.99%	11.47 (4.22–18.72)
10–14.99%	19.50 (1.6–28.4)

*p = 0.002*.

**Table 3:** Dose response relationship between fluid overload and oxygenation index.

**Total FO**	**Regression coefficient**	***p*-value**
<5%	0.29	0.15
5–9.99%	0.2	0.07
10–14.99%	0.31	<0.01
>15%	0.33	<0.02

**Table 4:** Maximum fluid overload and PELOD score as predictors of peak oxygenation index.

**Parameter**	**Peak oxygenation index**
Maximum FO	*r* = 0.38 (0.04–0.46); *p* = 0.04
PELOD	*r* = 0.18 (0.07–0.28); *p* = 0.002

### PICU survivors vs. non-survivors

Children who died had a higher admission PELOD score, higher peak FO% and maximum OI (Table 5). On multivariable analysis, both FO% and maximum OI were independent predictors of mortality.

**Table 5:** Mortality statistics of PICU patients.

	**Survivors (*n* = 69)**	**Non-survivors (*n* = 33)**	***p*-value**
Age (years)	6.15 ± 5.50	7.26 ± 6.82	0.38
Ventilation (days)	5.42 ± 3.62	6.5 ± 6.87	0.3
ICU stay (days)	8.36 ± 4.79	10.72 ± 12.60	0.17
PELOD Score at admission	12.50 ± 9.75	18.30 ± 12.10	0.01
Maximum FO %	7.11 ± 5.43	12.19 ± 11.26	0.002
Maximum oxygenation index	6.37 ± 5.12	16.83 ± 15.51	0.0001

### Fluid overload predicts mortality

Peak FO% was independently associated with mortality, controlled for age, PELOD score, and gender, even when the FO% was divided into groups 5–9.9%, 10–14.99% and >15%. At peak FO%>15%, the adjusted OR for mortality was 3.675 (95% CI 0.28–23.18; *p* = 0.039) (Table 6). We could not find any association between age and mortality, even after dividing age among sub-groups.

**Table 6:** Independent association of peak FO with mortality.

	**Parameter**	**Adjusted OR for mortality (95% CI)**	***p*-value**
Peak FO%	<5% (Baseline/Constant)		
	5–9.99%	2.520 (1.80–7.92)	0.011
	10–14.99%	2.751 (1.32–15.66)	0.041
	>15%	3.675 (1.28–23.18)	0.039
Age groups	<1 year (Baseline/Constant)		
	1–3 years	0.173 (0.02–1.19)	0.075
	3–12 years	0.301 (0.06–1.50)	0.144
	12–18 years	1.471 (0.41–5.22)	0.551

### Fluid overload predicts the duration of ventilation

Peak FO was significantly associated with PELOD score at admission and ventilation days on both univariable analysis (Table 7). Peak FO% was independently associated with length of ventilation, when controlled for PELOD score, age, and respiratory diagnosis (HR 0.78; 95% CI 0.66–0.87, *p* = 0.04).

**Table 7:** Association of peak fluid overload and clinical parameters.

**Parameter**	**Regression coefficient (95% CI)**	***p*-value**
Age (years)	−0.234 (0.049 to 0.031)	0.082
Sex	−0.732 (−4.00 to 2.54)	0.658
PELOD Score at admission	0.219 (0.075 to 0.36)	0.003
Ventilation (days)	0.342 (0.17 to 0.85)	0.0388
ICU (days)	−0.029 (−0.46 to 0.40)	0.896
Hospital stay (days)	0.148 (−0.088 to 0.38)	0.215

### Renal angina index and prediction of acute kidney injury on day 3 of admission

Thirty-eight children had RAI > 8 at admission (37.2%). Thirty-three children had AKI on day 3 of admission (32.3%). Discrimination of RAI by fluid overload was found to be superior than RAI calculated by change in creatinine clearance or when worse parameter was taken into consideration (Table 8). RAI prediction of day-3 AKI was superior to PRISM score at admission with a better AUC.

**Table 8:** Value of renal angina index to predict AKI and mortality.

Renal Angina Index ≤8, *n* (%)	38 (37.2%)
AKI by Day 3, *n* (%)	33 (32.3%)
Sensitivity to predict Day 3 AKI, % (95% CI)	81.8 (67–91.9)
Specificity to predict Day 3 AKI, % (95% CI)	69.6 (62.5–74.4)
PPV to predict Day 3 AKI, % (95% CI)	56.3 (46.1–63.2)
NPV to predict Day 3 AKI, % (95% CI)	88.9 (79.8–95.0)
AUC for any RAI, % (95% CI)	0.73 (0.61–0.82)
AUC, RAI as change CrCl, % (95% CI)	0.62 (0.57–72)
AUC, RAI as Fluid overload, % (95% CI)	0.78 (0.59–0.88)
AUC, PRISM, % (95% CI)	0.66 (0.61–0.73)

*RAI, Renal Angina Index; PPV, Positive Predictive Value; NPV, Negative Predictive Value; AUC, Area Under Curve; PRISM, Pediatric Risk of Mortality Score*.

## Discussion

In this prospective study, we looked at fluid overload and oxygenation status in children, and also correlations of RAI. Previous studies done by Ayse et al. (retrospective chart review of 80 patients) (1) and Basu et al. (16, 19) were done on retrospective cohorts.

Our study showed that fluid overload occurs early during the ICU stay and is detrimental to oxygenation, ventilation and overall outcome of critically ill children. Peak fluid overload and daily cumulative fluid overload positively correlated with oxygenation index, and the strength of association increased with increasing fluid overload.

A similar pattern of early occurrence of fluid overload was shown by Ayse et al. in a retrospective chart review of 80 patients(1). Ayse et al., from Texas, showed in the retrospective chart review on critically ill children, that higher peak fluid overload percent in sick children predicts higher peak oxygenation index, independent of age, gender, and PELOD (*p* = 0.009). Fluid overload percent ≥15% on any given day in the intensive care was also independently associated with that day's oxygenation index (*p* < 0.05) (1).

In our study, fluid overload and PELOD scores determined oxygenation indices of the patients. ICU survivors had a low PELOD score at admission, were less fluid overloaded, and had a low oxygenation index, which was statistically significant. However, Ayse et al. could only show less fluid overload in the survivors in their retrospective review.

Fluid overloaded children were sick as per the daily PELOD scores and required more ventilation. Fluid overload was an independent predictor of mortality, and with increasing fluid overload, the adjusted odds ratio increased. Children who were more than 15% overloaded had higher odds of mortality. Thus the goal of >15% in critically sick children in ICU to start contemplating about decongestive therapy could be taken by the treating physician. The current guidelines for management of septic shock in children call for intervention for fluid removal beyond 10% fluid overload with diuretics or intra- or extracorporeal RRTs (22). There are multiple pediatric and adult studies supporting the evidence of fluid overload as an independent predictor of mortality and worse outcomes in critically ill patients (23, 24). Our study findings add to the evidence of an association of worsening OI with FO > 15%. It also adds to the potential physiologic rationale for using FO to guide RRT initiation irrespective of presence AKI or conservative fluid management in children.

One of the strengths of the present study is the increased prevalence of nervous system (27.5%), hepatic (23.5%), and septic (15.7%) dysfunction in the prospective cohort. This is unlike Ayse et al. study where primary respiratory diagnosis was seen in 62.5% (1). Finding the adverse effects of FO and ventilation and outcomes in a non-respiratory group adds more evidence to this rationale. We chose not to include children on RRT to demonstrate the association in a general PICU population.

Our study shows that the RAI is better than conventional PRISM scoring for predicting AKI on day 3 of admission. The prediction when RAI was calculated as per fluid overload was better than when calculated as per change in creatinine. Basu et al. have showed this in a previous cohort study (16). In the multicenter four cohort appraisal, Basu et al. showed that the incidence rates for a Day 0 RAI of 8 or more were 15–68% and Day-3 AKI was 13-21% respectively. In all cohorts, Day-3 AKI rates was higher in patients who had an RAI of 8 or more with the area under the curve of RAI for predicting Day-3 AKI of 0.74–0.81. An RAI under 8 had very high negative predictive values (92–99%) for Day-3 AKI. They showed that RAI outperformed traditional markers of pediatric severity of illness (Pediatric Risk of Mortality-II) and AKI risk factors alone for prediction of Day-3 AKI. They also showed that, the RAI outperformed all KDIGO stages for prediction of Day-3 AKI (16).

The negative predictive value of 89% in our study population indicates that Day 0 ANG(–) patients have a very low likelihood of having a AKI or prolonged oliguria on Day 3 of ICU stay. This data may suggest that AKI biomarkers should not be obtained to predict AKI in Day 0 ANG(–) children, given the chances of Day-3 AKI, and will be worthwhile for clinicians taking care of critically sick children.

The RAI is easy to perform and can be done at bedside in the PICU. Identification of patients at a higher AKI risk using RAI stratification could theoretically guide the enrollment for a novel AKI biomarker or therapy trial, which could ultimately guide treatment strategy (19). Moreover, this can help physicians in judicious fluid and drug management in these patients. We feel that RAI should also be done in all critically ill children along with illness severity scores at the time of admission. One of the limitations of this study was our use of emergency department admission creatinine and fluid balance as proxies for their baseline values. Further, the fluid balance of the child in PICU does not include daily fluid losses during ventilation due to the difficulty of such measurements. In the clinic, electrical bioimpedence measurements may help the intensivist as a correlate of daily fluid shifts in these patients (25–27).

A limitation of our study is that serum creatinine was not corrected as per the fluid overload status of the child, and hence maybe more children have AKI, and missed due to the fluid imbalance. Future studies may help delineate whether FO truly is a causative factor in oxygenation failure and outcome. A daily bedside assessment of fluid balance should now be taken as a vital parameter.

## Conclusions

This study emphasizes that positive fluid balance adversely affects the ICU course in critically ill children. The RAI prediction model may further help optimize treatment and predict AKI.

## Ethics statement

This study was carried out in accordance with the recommendations of IRB committee, at Medanta with written informed consent from all subjects. All subjects gave written informed consent in accordance with the Declaration of Helsinki. The protocol was approved by the IRB Committee, Medanta, The Medicity.

## Author contribution

All authors listed have made a substantial, direct and intellectual contribution to the work, and approved it for publication.

### Conflict of interest statement

The authors declare that the research was conducted in the absence of any commercial or financial relationships that could be construed as a potential conflict of interest.

## References

[1] Arikan AA Zappitelli M Goldstein SL Naipaul A Jefferson LS Loftis LL. Fluid overload is associated with impaired oxygenation and morbidity in critically ill children. Pediatr Crit Care Med. (2012) 13:253–8. 10.1097/PCC.0b013e31822882a3 21760565

[2] Goldstein SL Currier H Graf Cd Cosio CC Brewer ED Sachdeva R. Outcome in children receiving continuous venovenous hemofiltration. Pediatrics (2001) 107:1309–12. 10.1542/peds.107.6.1309 11389248

[3] Goldstein SL Somers MJ Baum MA Symons JM Brophy PD Blowey D. Pediatric patients with multi-organ dysfunction syndrome receiving continuous renal replacement therapy. Kidney Int. (2005) 67:653–8. 10.1111/j.1523-1755.2005.67121.x 15673313

[4] Sutherland SM Zappitelli M Alexander SR Chua AN Brophy PD Bunchman TE. Fluid overload and mortality in children receiving continuous renal replacement therapy: the prospective pediatric continuous renal replacement therapy registry. Am J Kidney Dis. (2010) 55:316–25. 10.1053/j.ajkd.2009.10.048 20042260

[5] Selewski DT Cornell TT Lombel RM Blatt NB Han YY Mottes T. Weight-based determination of fluid overload status and mortality in pediatric intensive care unit patients requiring continuous renal replacement therapy. Intensive Care Med. (2011) 37:1166–73. 10.1007/s00134-011-2231-3 21533569 PMC3315181

[6] Valentine SL Sapru A Higgerson RA Spinella PC Flori HR Graham DA. Fluid balance in critically ill children with acute lung injury. Crit Care Med. (2012) 40:2883–9. 10.1097/CCM.0b013e31825bc54d 22824936 PMC3455114

[7] Hazle MA Gajarski RJ Yu S Donohue J Blatt NB. Fluid overload in infants following congenital heart surgery. Pediatr Crit Care Med. (2013) 14:44–9. 10.1097/PCC.0b013e3182712799 23249789 PMC3668443

[8] Bouchard J Soroko SB Chertow GM Himmelfarb J Ikizler TA Paganini EP. Fluid accumulation, survival and recovery of kidney function in critically ill patients with acute kidney injury. Kidney Int. (2009) 76:422–7. 10.1038/ki.2009.159 19436332

[9] Payen D de Pont AC Sakr Y Spies C Reinhart K Vincent JL A positive fluid balance is associated with a worse outcome in patients with acute renal failure. Crit Care (2008) 12:R74 10.1186/cc6916 18533029 PMC2481469

[10] Murphy CV Schramm GE Doherty JA Reichley RM Gajic O Afessa B. The importance of fluid management in acute lung injury secondary to septic shock. Chest (2009) 136:102–9. 10.1378/chest.08-2706 19318675

[11] Heung M Wolfgram DF Kommareddi M Hu Y Song PX Ojo AO. Fluid overload at initiation of renal replacement therapy is associated with lack of renal recovery in patients with acute kidney injury. Nephrol Dial Transplant. (2012) 27:956–61. 10.1093/ndt/gfr470 21856761 PMC3471547

[12] Mitchell JP Schuller D Calandrino FS Schuster DP. Improved outcome based on fluid management in critically ill patients requiring pulmonary artery catheterization. Am Rev Respir Dis. (1992) 145:990–8. 10.1164/ajrccm/145.5.990 1586077

[13] Grams ME Estrella MM Coresh J Brower RG Liu KD. Fluid balance, diuretic use, and mortality in acute kidney injury. Clin J Am Soc Nephrol. (2011) 6:966–73. 10.2215/CJN.08781010 21393482 PMC3087792

[14] Goldstein S Bagshaw S Cecconi M Okusa M Wang H Kellum J. Pharmacological management of fluid overload. Br J Anaesth. (2014) 113:756–63. 10.1093/bja/aeu299 25209097

[15] Wiedemann HP Wheeler AP Bernard GR. Comparison of two fluid-management strategies in acute lung injury. N Engl J Med. (2006) 354:2564–75. 10.1056/NEJMoa062200 16714767

[16] Basu RK Kaddourah A Terrell T Mottes T Arnold P Jacobs J. Assessment of Worldwide Acute Kidney Injury, Renal Angina and Epidemiology in critically ill children (AWARE): study protocol for a prospective observational study. BMC Nephrol. (2015) 16:24. 10.1186/s12882-015-0016-6 25882434 PMC4355130

[17] Brandstrup B Tønnesen H Beier-Holgersen R Hjortsø E Ørding H Lindorff-Larsen K. Effects of intravenous fluid restriction on postoperative complications: Comparison of two perioperative fluid regimens: a randomized assessor-blinded multicenter trial. Ann Surg. (2003) 238:641–8. 10.1097/01.sla.0000094387.50865.23 14578723 PMC1356139

[18] Schuller D Mitchell JP Calandrino FS Schuster DP Fluid balance during pulmonary edema. Is fluid gain a marker or a cause of poor outcome? Chest (1991) 100:1068–75. 1914560 10.1378/chest.100.4.1068

[19] Basu RK Wang Y Wong HR Chawla LS Wheeler DS Goldstein SL. Incorporation of biomarkers with the renal angina index for prediction of severe AKI in critically ill children. Clin J Am Soc Nephrol. (2014) 9:654–62. 10.2215/CJN.09720913 24677554 PMC3974366

[20] Basu RK Zappitelli M Brunner L Wang Y Wong HR Chawla LS. Derivation and validation of the renal angina index to improve the prediction of acute kidney injury in critically ill children. Kidney Int. (2014) 85:659–67. 10.1038/ki.2013.349 24048379 PMC4659420

[21] Upadya A Tilluckdharry L Muralidharan V Amoateng-Adjepong Y Manthous CA. Fluid balance and weaning outcomes. Intensive Care Med (2005) 31:1643–7. 10.1007/s00134-005-2801-3 16193330

[22] Brierley J Carcillo JA Choong K. Clinical practice parameters for hemodynamic support of pediatric and neonatal septic shock: 2007 update from the American College of Critical Care Medicine. Crit Care Med. (2009) 37:666–88. 10.1097/CCM.0b013e31819323c6 19325359 PMC4447433

[23] Gillespie RS Seidel K Symons JM. Effect of fluid overload and dose of replacement fluid on survival in hemofiltration. Pediatr Nephrol. (2004) 19:1394–1399. 10.1007/s00467-004-1655-1 15517417

[24] Hayes LW Oster RA Tofil NM Tolwani AJ. Outcomes of critically ill children requiring continuous renal replacement therapy. J Crit Care (2009) 24:394–400. 10.1016/j.jcrc.2008.12.017 19327959

[25] Newman RB Pierre H Scardo J. Thoracic fluid conductivity in peripartum women with pulmonary edema. Obstet Gynecol. (1999) 94:48–51. 10389716 10.1016/s0029-7844(99)00232-x

[26] Saunders CE. The use of transthoracic electrical bioimpedance in assessing thoracic fluid status in emergency department patients. Am J Emerg Med. (1988) 6:337–340. 3390250 10.1016/0735-6757(88)90151-9

[27] Underwood MJ Pearson JA Waggoner J Lunec J Firmin RK Elliot MJ. Changes in “inflammatory” mediators and total body water during extra-corporeal membrane oxygenation (ECMO). A preliminary study. Int J Artif Organs (1995) 18:627–63. 8647596

